# Single-Item Assessment of Quality of Life: Associations with Well-Being, Mood, Health Correlates, and Lifestyle

**DOI:** 10.3390/jcm13175217

**Published:** 2024-09-03

**Authors:** Joris C. Verster, Emina Išerić, Guusje A. Ulijn, Stephanie M. P. Oskam, Johan Garssen

**Affiliations:** 1 Division of Pharmacology, Utrecht Institute for Pharmaceutical Sciences, Utrecht University, 3584 CG Utrecht, The Netherlands; e.iseric@students.uu.nl (E.I.); g.a.ulijn@students.uu.nl (G.A.U.); s.m.p.oskam@students.uu.nl (S.M.P.O.); j.garssen@uu.nl (J.G.); 2 Centre for Mental Health and Brain Sciences, Swinburne University, Melbourne, VIC 3122, Australia; 3 Cognitive Neurophysiology, Department of Child and Adolescent Psychiatry, Faculty of Medicine, TU Dresden, D-01307 Dresden, Germany; 4 Danone Global Research & Innovation Center, Uppsalalaan 12, 3584 CT Utrecht, The Netherlands

**Keywords:** quality of life, single-item assessment, mood, immune fitness, sleep, well-being, nutrition, stress, lifestyle

## Abstract

**Background:** Quality of life (QoL) is traditionally assessed using multiple-item questionnaires. These can be either general, global assessments of QoL or disease-specific questionnaires. However, the use of single-item QoL scales is becoming increasingly popular, as these are more time- and cost-effective, with a readily available and easy-to-interpret outcome. In particular, these are often preferred for quick assessments (e.g., ‘at home’ testing and mobile phone assessments), and other cases when time constraints are common (e.g., clinical trials and clinical practice). Previous research revealed that multiple-item questionnaires and single-item assessments of QoL have the same validity and reliability. Here we further evaluate the relationship of QoL, assessed with a single-item QoL scale, with well-being, mood, health correlates (e.g., immune fitness, and having underlying diseases), and lifestyle (e.g., sleep, nutrition). **Methods:** Data from two online surveys are presented. In Study 1, 100 students participated. The single-item QoL score was compared with the World Health Organization Well-Being Index (WHO-5), a single-item score of sleep quality, the Regensburg Insomnia Scale (RIS) score, and the Healthy Diet Scale (HDS). Study 2 comprised a survey among 1415 Dutch adults. Single-item QoL was evaluated and compared with assessments of mood, health correlates (immune fitness and disease status), and lifestyle factors (e.g., sleep, nutrition, stress). **Results:** The first study revealed significant correlations between QoL and well-being, sleep quality, insomnia ratings, and attaining a healthy diet. The second study revealed significant correlations between QoL and mood, health status, and lifestyle factors (e.g., the ability to cope with stress). **Conclusions:** The results presented here demonstrate that the single-item QoL scale is an effective and easy-to-implement assessment tool that can be used in both clinical practice and research.

## 1. Introduction

The World Health Organization (WHO) defines quality of life (QoL) as “an individual’s perception of their position in life in the context of the culture and value systems in which they live and in relation to their goals, expectations, standards and concerns” [1]. QoL is a subjective evaluation that is embedded in a cultural, social, and environmental context [1]. QoL is a crucial concept in health and disease [2,3], as its perception can motivate individuals to maintain or adjust lifestyle and health behaviors. For clinicians and researchers, assessment of QoL provides an important indicator of the impact on health status and interventions on a patient’s daily life.

There are numerous scales to assess QoL [4], and these can be either general QoL assessments (unrelated to the patient’s condition) or disease-specific QoL scales. Traditionally, these scales are multiple-item questionnaires, which can be elaborate. For example, the WHOQOL-100 comprises 100 items [5]. Thus, it can be time-consuming to complete such scales. The acceptability of assessment tools by patients is crucial. Lengthy questionnaires may be a burden to certain populations (e.g., the elderly or patients with severe sickness) and result in reduced completion rates. Also, in survey research, it is known that longer questionnaires result in greater dropouts of participants. With regard to methodology, the most important advantage of a single-item global assessment of a concept (in this case, QoL) is that this single-score approach evaluates the entire constellation of what constitutes the concept, instead of limiting the concept to the sum of the selected items that are part of a multiple-item questionnaire [6,7].

The 2019 coronavirus disease (COVID-19) pandemic also pointed at the need for alternative, shorter measures of QoL [8]. The associated lockdowns and restrictions had a significant impact on the conductance of clinical trials, making it harder to do in-person assessments. Alternative trial designs were developed, including ‘at home’ testing and ‘mobile’ assessments on phones and tablets [8]. Instead of clinical interviews and multiple-item questionnaires, short single-item scales became more popular and showed similar validity and reliability [9]. An example of such single-item scales is the 11-point mood scale, ranging from absent (score 0) to extreme (score 10), to assess mood items such as stress, anxiety, and depression [6,10]. The single-item mood scales were shown to be equally valid and reliable as their corresponding multiple-item questionnaires [6,10]. A single-item scale was also developed to assess immune fitness, i.e., the body’s capacity to respond to health challenges (such as infections) by activating an appropriate immune response, which is essential to maintain health and prevent disease [11]. Immune fitness was measured on an 11-point scale ranging from very poor (score 0) to excellent (score 10). In addition, similar single-item rating scales were developed to assess sleep quality [12], attaining a healthy diet [13], and QoL.

Already 40 years ago, single-item QoL scales were developed [14]. Direct comparisons with multiple-item scales showed the advantages of single-item QoL scales, such as being more time- and cost-effective, while being equally valid and reliable as multiple-item assessments [15]. For example, it was shown that a single-item assessment of QoL correlated significantly with a multiple-item QoL scale, the Short Form General Health Survey of the Medical Outcomes Study (MOS) [16]. Thus, the use of a single-item scale to assess QoL is not new. For example, De Boer et al. [17] developed and tested a single-item QoL scale, ranging from 0 to 100. The single-item QoL scale was tested in 83 patients with esophageal adenocarcinoma. Significant correlations were found between QoL with the multi-item Medical Outcomes Study Short Form-20 (MOS SF-20), and between QoL and the Rotterdam Symptom Checklist. A test–retest reliability intra-class correlation of 0.87 was found. Taken together, the single-item QoL scale had good validity and excellent reliability. Siebens et al. [18] used an alternative single-item scale to assess QoL. The question “Taking everything in your life into account, please rate your overall quality of life on the following seven-point scale” was scored on a seven-point scale ranging from “Life is very distressing” to “Life is great”. Data were collected from patients with cerebral palsy, polio, rheumatoid arthritis, and stroke. Low QoL was associated with lower functional levels and higher depression scores, whereas a greater level of social interactions was associated with better QoL.

**Figure 1:**
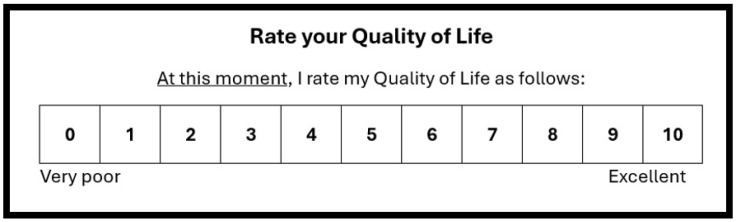
The single-item assessment of quality of life.

Previous research including 108 Dutch young adults (with a mean age of 21.5 years old (28.7% males)) also evaluated the reliability of a single-item QoL scale [10]. The 11-point single-item QoL scale used in this study ranges from very poor (score 0) to excellent (score 10) (see Figure 1). On the same day, a test–retest assessment was conducted, with only 30 min between the test sessions. Although there was a short time period between the measures, participants were unaware that the second assessment would take place. The analysis confirmed that the single-item QoL scale has good reliability.

First, no significant difference was found between the mean (SD) of the test (7.52 (1.0)) and retest assessment (7.41 (1.0)). Second, the Pearson’s correlation between the test and retest assessment was significant (r = 0.674, *p* < 0.001). Third, the intraclass correlation of 0.672 (95%CI lower and upper limits: 0.555 and 0.764, respectively) suggests moderate to good agreement between the test and retest assessment. Finally, the Bland–Altman limits of agreement analysis concluded agreement between the test and re-test assessment: a mean (SD) difference of 0.11 (0.8) between the test and retest agreement (95% CI: −1.68, 1.46) with only 2.8% of assessments outside the limits of agreement interval. Thus, when adopting an ultra-short test–retest period, the single-item QoL scale still had good reliability.

In conclusion, single-item QoL scales exhibit adequate validity and reliability, and they have been utilized in various studies. The primary aim of Study 1 was to compare the single-item QoL scale with the 5-item World Health Organization Well-Being Index (WHO-5) [19]. In addition, immune fitness, sleep, and daily diet were assessed as factors that are potentially related to QoL. The aim of Study 2 was to further evaluate the relationship of QoL with mood, health correlates (e.g., immune fitness, having underlying diseases), and lifestyle (e.g., sleep, nutrition).

## 2. Materials and Methods

Data from two studies were used to analyze the relationship between QoL and well-being (Study 1) as well as between QoL and mood, health correlates, and lifestyle (Study 2).

### 2.1. Study 1

Survey data from an online study among 101 Dutch university students was used to evaluate the association between QoL and well-being. For this comparison, a sample size >100 is considered excellent [20]. Informed consent was obtained from all participants, and the study was approved by the Science-Geo Ethics Review Board of Utrecht University (approval code: S-23040, approval date: 27 June 2023). In this survey, the single-item QoL scale was completed. On the 11-point single-item QoL scale, participants rated their quality of life on a scale ranging from very poor (score 0) to excellent (score 10). Recorded demographic data included age, sex, bodyweight, and academic year (year 1, 2, or 3). In addition, immune fitness was assessed on a scale ranging from 0 (very poor) to 10 (excellent) [11].

Participants completed the WHO-5 [19]. The WHO-5 is a questionnaire consisting of 5 questions, used to assess subjective psychological well-being. The six answer possibilities include “at no time” (score 0), “some of the time” (score 1), “less than half of the time” (score 2), “more than half of the time” (score 3), “most of the time” (score 4), and “all of the time” (score 5). The participants chose the answer closest to how they had been feeling over the past 6 months [19]. The raw WHO-5 sum score ranges from 0 to 25 and is multiplied by 4 to yield a final score ranging from 0 (worst imaginable well-being) to 100 (best imaginable well-being).

Sleep quality was assessed with a single-item scale, ranging from 0 (very poor) to 10 (excellent) [12]. Insomnia was assessed with the 10-item Regensburg Insomnia Scale (RIS) [21]. The RIS items cover quantitative and qualitative sleep parameters (e.g., sleep latency and total sleep time) and psychological aspects of insomnia, such as fear of insomnia and daytime fitness. Items are scored on a 5-point Likert scale, and the total score ranges from 0 to 40 points, with higher scores indicating greater insomnia complaints. The RIS has a Cronbach alpha of 0.890, indicating good reliability.

To what extent participants attain a healthy diet was assessed with the single-item Healthy Diet Scale [13]. Participants could rate the percentage of their daily diet they considered healthy on an 11-point scale ranging from 0% (unhealthy) to 100% (healthy). In this study, we used the Healthy Diet Scale without food examples of what constitutes a healthy diet. Previous research revealed that the Healthy Diet Scale with and without food examples yielded comparable results [22]. In Study 1, all assessments were made retrospectively for the past 6 months.

### 2.2. Study 2

Data from 1415 Dutch adults who completed an online survey was used to evaluate the relationship between QoL and mood and health [23]. Informed consent was obtained from all participants, and the study was approved by the Ethics Committee of the Faculty of Social and Behavioral Sciences of Utrecht University (approval code: FETC17-061; approval date: 8 June 2017).

Participants completed retrospective assessments for the period January 1st–March 15th, 2020 (i.e., the period before the start of the 2019 coronavirus disease pandemic in the Netherlands). Recorded demographic data included age, sex, body mass index (BMI), and education level (low, medium, and high). The single-item QoL scale was incorporated in the survey. Mood was assessed using single-item ratings ranging from absent (score 0) to extreme (score 10) and included the items stress, anxiety, depression, fatigue, hostility, loneliness, and happiness [6,10].

Health status was reflected by the assessment of immune fitness and the reported number of chronic health conditions. Immune fitness was assessed with a single-item scale ranging from 0 (poor) to 10 (excellent) [6,11]. Participants could further indicate whether they had one or more common chronic medical conditions [24], including cardiovascular diseases or hypertension, diabetes, liver disease, neurological diseases, immune disorders, allergy, kidney disease, pulmonary diseases, anxiety, depression, sleep disorders, or “other”. The number of reported chronic diseases and conditions was used for the current analysis. A detailed discussion of the study methodology has been published elsewhere [23].

A subsample of 514 participants also completed a follow-up survey assessing lifestyle [22]. Lifestyle factors were assessed with a modified version of the FANTASTIC Lifestyle Checklist [25,26,27]. The modified checklist comprised 16 questions (see Table 1) that assess the lifestyle factors (1) support of family and friends, (2) physical activity level, (3) nutrition, (4) tobacco and toxins, (5) sleep, (6) coping with stress, (7) optimism, and (8) role satisfaction. Each question had 5 answering possibilities (see the note in Table 1 for specific scoring instructions). For each lifestyle factor, the sum of item scores was computed. Higher scores on the lifestyle factors represent a better and/or healthier lifestyle.

### 2.3. Statistical Analysis

The statistical analyses were conducted with SPSS (IBM Corp. Released 2013. IBM SPSS Statistics for Windows, Version 29.0. IBM Corp., Armonk, NY, USA). The mean, median, interquartile range, and standard deviation (SD) were computed for all variables. Normal distribution of the outcome measures was determined via visual inspection and the Kolmogorov–Smirnov test. As it appeared that most data were not normally distributed, nonparametric statistical tests were used for the presented analyses.

To evaluate the relationship between QoL and well-being (Study 1), Spearman’s correlations were computed between the single-item QoL scale and the WHO-5 total score (*p* < 0.05 for significance) and between the single-item QoL scale and individual WHO-5 items (*p* < 0.01 for significance, after Bonferroni’s correction). To evaluate relationships between the single-item QoL scale score and assessments of mood, health, and lifestyle (Study 2), Spearman’s correlations were computed (*p* < 0.05 for significance). A Bonferroni’s correction was applied for multiple correlations with mood items (*p* < 0.007 for significance) and lifestyle (*p* < 0.00625 for significance). Differences in QoL scores between groups with and without underlying disease were evaluated with the Independent-Samples Kruskal–Wallis test, applying a Bonferroni’s correction for multiple comparisons (*p* < 0.01 for significance). Finally, stepwise linear regression analysis was conducted to identify variables that significantly predict QoL. Variables included in the analyses were age, sex, BMI, education level, underlying disease status, immune fitness, mood items, and lifestyle factors.

## 3. Results

### 3.1. Study 1

In this study, *n* = 101 Dutch students participated. Their mean (SD) age was 20.4 (1.8) years old, and 17% were males. They had a mean (SD) bodyweight of 65.9 (11.8) kg and were attending academic year 1 (31.8%), 2 (29.5%), or 3 (38.6%). The mean (SD) QoL score was 7.1 (1.3) and did not differ significantly between males and females (*p* = 0.328). No significant correlations were found between QoL and age, bodyweight, and academic year. A significant positive correlation was found between QoL and immune fitness (r = 0.341, *p* < 0.001).

Validity of the single-item QoL scale was assessed by comparing its outcome with the WHO-5. A significant Spearman’s correlation was found between the single-item QoL scale and the WHO-5 (r = 0.534, *p* < 0.001). All individual items of the WHO-5 correlated significantly with the single-item QoL scale (see Table 2).

The participants reported a mean (SD) sleep quality of 6.85 (1.4), with a range from 3 to 10. A significant positive correlation was found between QoL and sleep quality (r = 0.585, *p* < 0.001). A significant negative correlation was found between QoL and the RIS insomnia score (r = −0.428, *p* < 0.001), indicating that having more insomnia complaints is associated with poorer QoL. Spearman’s correlations with the individual RIS insomnia items are summarized in Table 3. Significant correlations were found between QoL and items related to sleep quality, such as “my sleep is disturbed” and “I feel that I have not slept all night,” and with items related to rumination and worrying about sleep (e.g., “I think a lot about my sleep”). The strongest correlation was found between QoL and the daytime consequences of poor sleep (i.e., “I feel fit during the day”). Taken together, sleep has a significant impact on QoL.

With regard to daily diet, participants reported that they considered 64.2% of their daily diet as healthy (SD = 14.1%). A significant and positive correlation was found between attaining a healthy diet and QoL (r = 0.323, *p* < 0.001).

In conclusion, a significant and positive correlation was found between QoL and the WHO-5. In addition, significant correlations were found between QoL and sleep and QoL and daily diet. As these factors potentially influence the relationship between QoL and the WHO-5, a partial correlation was computed. The partial correlation between QoL and the WHO-5, corrected for sleep, immune fitness, and daily diet, remained significant (r = 0.465, *p* < 0.001).

### 3.2. Study 2

Data from 1415 Dutch adults (64.5% female) were considered for the analysis. Their mean (SD) age was 45.0 (18.5) years old (range of 18 to 94). They had a mean (SD) BMI of 26.5 (5.8) kg/m^2^, a mean (SD) immune fitness score of 7.3 (1.9), and their education level was either low (36.4%), medium (26.3%), or high (37.3%). The mean (SD) QoL score was 7.18 (2.2). No significant difference in QoL was found between men (mean (SD): 7.2 (2.2)) and women (mean (SD): 7.1 (2.1)). There was no significant correlation between QoL and age (r = 0.008, *p* = 0.766). Low but statistically significant correlations were found between QoL and BMI (r = −0.059, *p* = 0.025), and between QoL and education level (r = −0.066, *p* = 0.013).

With regard to health, a positive and significant correlation was found between QoL and immune fitness (r = 0.379, *p* < 0.001). Thus, a better self-reported body’s capacity to respond to health challenges (such as infections) by activating an appropriate immune response was associated with greater quality of life.

**Figure 2:**
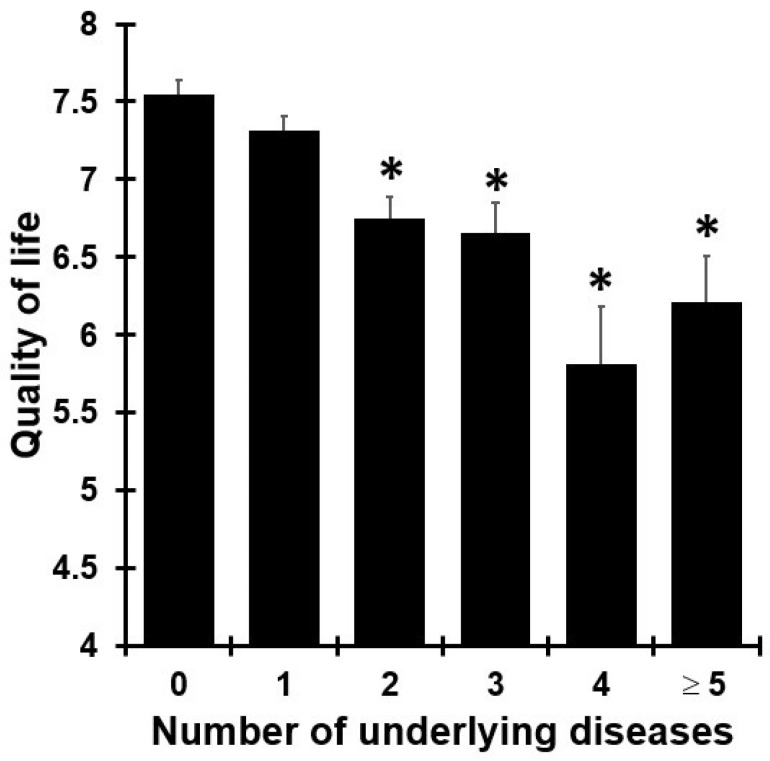
Quality of life and underlying disease. Results are shown from *n* = 1415 Dutch adults [23]. Differences in quality of life, compared to the group without underlying diseases, were considered statistically significant if *p* < 0.01 (after Bonferroni’s correction) and indicated with *.

A total of *n* = 920 participants (65.5% of the sample) reported having one or more underlying medical conditions. Most frequently reported were allergy (35.0%), cardiovascular diseases or hypertension (25%), sleep disorders (20.4%), pulmonary diseases (19.1%), and depression (16.0%). A significant relationship was also found between the number of reported underlying diseases and quality of life (r = −0.212, *p* < 0.001). Figure 2 shows that QoL is significantly decreased among participants who report a combination of two or more underlying diseases.

The Spearman’s correlations of QoL with mood outcomes are summarized in Table 4. All mood items correlated significantly and negatively with QoL, except for the robust positive correlation between QoL and happiness.

A subsample of 514 participants (35.8% males, with a mean (SD) age of 44.8 (19.0) years old) also completed questions on lifestyle. Spearman’s correlations of lifestyle with QoL are summarized in Table 5. Significant positive correlations were found, indicating that greater support of family and friends, higher levels of physical activity, better sleep quality, better coping with stress, and higher levels of optimism and role-satisfaction were associated with a better QoL. Interestingly, the use of tobacco and toxins was significantly associated with a better QoL. Thus, although there is a modest correlation, according to the participants, the use of tobacco, alcohol, drugs, and caffeine to some extent contributes positively to their QoL.

#### Predictors of QoL

A stepwise regression analysis was conducted to identify significant predictors of QoL. The variables included were demographics (sex, age, BMI, and education level), health status (having underlying diseases and immune fitness), mood (stress, anxiety, depression, fatigue, hostility, loneliness, and happiness), and lifestyle factors (support of family and friends, physical activity level, nutrition, tobacco and toxins, sleep, coping with stress, optimism, and role-satisfaction). The analysis revealed a significant model (F_(5,504)_ = 93.08, *p* < 0.001) explaining 47.5% of variance in QoL (see Table 6). The five variables that were significant predictors of QoL were happiness (40.4%), depression (3.4%), immune fitness (1.7%), sex (1.1%), and sleep (0.9%).

## 4. Discussion

The results presented here demonstrate that the single-item QoL scale is an effective and easy-to-implement assessment tool that can be used in both clinical practice and research. The single-item QoL scale can be used for momentary assessments or retrospectively for any given time period. Its completion has minimal burden for patients, and the outcome is directly available.

The study outcomes revealed that the single-item QoL assessments correlated significantly with well-being, mood, sleep, immune fitness, and lifestyle factors such as the ability to cope with stress. These findings are in line with previous research using multiple-item questionnaires to assess QoL, which also showed that a better QoL is associated with better physical and mental health [2,3,4,26,27], and that adequate immune fitness and various lifestyle factors such as attaining a healthy diet, adequate sleep, and better coping with stress can improve QoL [28].

The single-item assessment is a global general assessment of QoL. The advantage of using traditional multiple-item QoL questionnaires is that the items assess many different aspects of QoL, or specific diseases, and are thus more informative than single-item QoL scales. However, the disadvantage of multiple-item QoL questionnaires is that it may be a burden to complete them for certain populations (e.g., the elderly). Given their relatively long completion time, it is not always possible to implement these assessments in clinical trials and clinical practice where time constraints are common. The current article demonstrates that in those instances, a single-item QoL scale may be a suitable alternative. From a theoretical perspective, single-item assessments may even be preferred above multiple-item scales [6,7,11]. When judging a concept such as anxiety, depression, or sleep quality, individuals weigh the characteristics and impact of the related complaints they experience. This is also applicable to the assessment of QoL. Individuals consider features such as the type of complaint, the number of experienced complaints, their frequency of occurrence, their severity and duration, and to what extent the complaints impact daily activities and interactions with others [11]. It is hypothesized that single-item assessments automatically incorporate all of these features [6,7,11]. In contrast, multiple-item scales per definition make a selection of these features and thus usually do not include all of them. Therefore, single-item scales provide a more holistic assessment compared to multiple-item scales. On the other hand, QoL is a multidimensional construct that can be interpreted (and therefore scored) differently between individuals. In both studies, no definition of QoL was provided along with the rating scale. This may have influenced the study outcome. Therefore, future studies should compare scoring of the QoL scale with and without a definition.

Limitations of Study 1 comprise its relatively small sample size and the fact that the population under investigation was limited to young, healthy adults. Nevertheless, robust correlations were found between QoL and well-being, sleep, and attaining a healthy diet. The strengths of Study 2 comprised its much larger sample size and the broad age range of the participants. The study aimed to cover the Dutch general adult population, and therefore both participants with and without underlying diseases completed the survey. In both surveys, females were overrepresented. Bot sex and age may have impacted the evaluated correlations with QoL.

As both studies collected data retrospectively, future research should further evaluate the usefulness of the single-item QoL scale in prospective studies, clinical trials, and intervention studies. In the currently presented data, all assessments relied on self-report. This was necessary, as there are no biomarkers or other objective measures for QoL and mood. At present, self-report is the only way to assess mood and QoL. For other measures (e.g., sleep and immune functioning), objective measures are available (polysomnography and biomarkers, respectively). In future studies, assessments of objective measures should be implemented to further support the currently observed relationships between self-reported assessments and QoL.

Some participants reported a positive relationship between poor health behaviors (i.e., smoking and alcohol consumption) and QoL. This could be due to the fact that some people cope with stress by consuming these products. Better coping with stress is correlated with better QoL. Also, the way ‘coping with stress’ is formulated in the questionnaire (i.e., ‘I relax and enjoy leisure time’) may include the use of tobacco and toxins for part of the study sample. Notwithstanding this, a clear negative correlation was found between the number of medical conditions reported and QoL.

The analyses revealed that several variables significantly correlated with QoL. In particular, the regression analysis identified happiness, depression, sleep, sex, and immune fitness as significant predictors of QoL. It should be noted that, in addition to being related to QoL, these factors also interact with each other. Therefore, future studies examining relationships of QoL with other variables should take into account demographics, lifestyle factors, mood, and immune fitness of the population under investigation and apply a statistical methodology to account for their interrelationship and impact on QoL.

In conclusion, significant correlations were found between QoL and well-being, mood, health correlates (i.e., immune fitness and having underlying diseases), and lifestyle (daily diet, sleep, coping with stress). Given this, the single-item QoL scale can be considered a useful assessment tool for both research and clinical practice.

## Figures and Tables

**Table 1:** The modified version of the FANTASTIC Lifestyle Checklist.

Lifestyle Factor	Question	Description
Support of family and friends	1	I have someone to talk to about things that are important to me
	2	I give and receive affection
Physical activity level	3	I am vigorously active for at least 30 min per day (e.g., running, cycling, etc.)
	4	I am moderately active (gardening, climbing stairs, walking, housework)
Nutrition	5	I eat a balanced diet
	6	I often eat excess (1) sugar, or (2) salt, or (3) animal fats, or (4) junk food
	7	I am within … kg of my healthy weight
Tobacco and toxins	8	I smoke tobacco
	9	I use drugs such as marijuana, cocaine
	10	I overuse prescribed or ‘over the counter’ drugs
	11	I drink caffeine containing coffee, tea or cola
Sleep	12	I sleep well and feel rested
Coping with stress	13	I am able to cope with the stresses in my life
	14	I relax and enjoy leisure time
Optimism	15	I am a positive or optimistic thinker
Role satisfaction	16	I am satisfied with my job or role

Note: Answering possibilities for questions 1, 2, 5, and 11–16 were ‘almost never’ (score of 0), ‘seldom’ (score of 1), ‘some of the time’ (score of 2), ‘fairly often’ (score of 3), or ‘almost always’ (score of 4). Answering possibilities for questions 3 and 4 were ‘less than once/week’ (score of 0), ‘1–2 times/week’ (score of 1), ‘3 times/week’ (score of 2), ‘4 times/week’ (score of 3), or ‘5 or more times/week’ (score of 4). Answering possibilities for question 6 were ‘four of these’ (score of 0), ‘three of these’ (score of 1), ‘two of these’ (score of 2), ‘one of these’ (score of 3), or ‘none of these’ (score of 4). Answering possibilities for question 7 were ‘not within 8 kg’ (score of 0), ‘8 kg’ (score of 1), ‘6 kg’ (score of 2), ‘4 kg’ (score of 3), or ‘2 kg’ (score of 4). Answering possibilities for question 8 were ‘more than 10 times/week’ (score of 0), ‘1–10 times/week’ (score of 1), ‘none in the past 6 months’ (score of 2), ‘none in the past year’ (score of 3), or ‘none in the past 5 years’ (score of 4). Answering possibilities for questions 9 and 10 were ‘almost daily’ (score of 0), ‘fairly often’ (score of 1), ‘only occasionally’ (score of 2), ‘almost never’ (score of 3), or ‘never’ (score of 4).

**Table 2:** Correlations of the single-item QoL scale with WHO-5 items.

WHO-5 Item	*r*	*p*-Value
I have felt cheerful and in good spirits	0.491	<0.001 *
I have felt calm and relaxed	0.489	<0.001 *
I have felt active and vigorous	0.433	<0.001 *
I woke up feeling fresh and rested	0.617	<0.001 *
My daily life has been filled with things that interest me	0.376	0.001 *
WHO-5 total score	0.568	<0.001 *

Spearman’s correlations between the single-item QoL scale and WHO-5 items were considered significant if *p* < 0.01 (after Bonferroni’s correction), and indicated with *. Abbreviations: WHO = World Health Organization; QoL = quality of life.

**Table 3:** Correlations of the single-item QoL scale with RIS insomnia items.

RIS Insomnia Items	*r*	*p*-Value
Sleep onset latency	−0.289	0.004 *
Total sleep time	−0.262	0.008
My sleep is disturbed	−0.426	<0.001 *
I wake up too early	−0.141	0.163
I wake up from the slightest sound	−0.180	0.074
I feel that I have not slept all night	−0.540	<0.001 *
I think a lot about my sleep	−0.371	<0.001 *
I am afraid to go to bed because of my disturbed sleep	−0.345	<0.001 *
I feel fit during the day	−0.609	<0.001 *
I take sleeping pills in order to get to sleep	−0.142	0.158

Spearman’s correlations between the single-item QoL scale with RIS insomnia items were considered significant if *p* < 0.005 (after Bonferroni’s correction), and indicated with *. Abbreviations: RIS = Regensburg Insomnia Scale; QoL = quality of life.

**Table 4:** Correlations between quality of life and mood.

Mood Items	*r*	*p*-Value
Stress	−0.274	<0.001 *
Anxiety	−0.321	<0.001 *
Depression	−0.398	<0.001 *
Fatigue	−0.277	<0.001 *
Hostility	−0.218	<0.001 *
Loneliness	−0.364	<0.001 *
Happiness	0.638	<0.001 *

Results are shown from *n* = 1415 Dutch adults [23]. Spearman’s correlations were considered statistically significant (after Bonferroni’s correction) if *p* < 0.007, and indicated with *.

**Table 5:** Correlations between quality of life and lifestyle.

Lifestyle Items	*r*	*p*-Value
Support of family and friends	0.259	<0.001 *
Physical activity level	0.149	<0.001 *
Nutrition	0.110	0.013
Tobacco and toxins	0.121	0.006 *
Sleep	0.403	<0.001 *
Coping with stress	0.410	<0.001 *
Optimism	0.414	<0.001 *
Role-satisfaction	0.353	<0.001 *

Results are shown from *n* = 514 Dutch adults [23]. Spearman’s correlations were considered statistically significant (after Bonferroni’s correction) if *p* < 0.00625, and indicated with *.

**Table 6:** Predictors of QoL.

RIS Insomnia Items	Adjusted R^2^	*β*	*t*	*p*-Value
Happiness	0.404	0.493	13.5	<0.001 *
Depression	0.438	−0.166	−4.6	<0.001 *
Immune fitness	0.455	0.121	3.5	<0.001 *
Sleep	0.464	0.130	3.6	<0.001 *
Sex	0.475	0.108	3.3	<0.001 *

Results of the stepwise regression analysis. *p*-values are significant if *p* < 0.05, indicated with *. Variables that did not significantly contribute to the model were age, BMI, education level, having underlying diseases, stress, anxiety, hostility, loneliness, support of family and friends, physical activity level, nutrition, tobacco and toxins, coping with stress, optimism, and role-satisfaction.

## Data Availability

The data are available upon reasonable request from the corresponding author.
